# Cisplatin-Containing Combinations Associate with Survival in Women from Appalachian Kentucky with Metastatic, Persistent, or Recurrent Uterine Cervix Cancer

**DOI:** 10.3390/cancers16193319

**Published:** 2024-09-28

**Authors:** Charles A. Kunos, Rachel W. Miller, Denise Fabian

**Affiliations:** 1 Department of Radiation Medicine, University of Kentucky, Lexington, KY 40508, USA; 2 Department of Obstetrics & Gynecology, Division of Gynecologic Oncology, University of Kentucky, Lexington, KY 40508, USA

**Keywords:** cervix, cervical cancer, Appalachian, metastatic, persistent, recurrent, cisplatin, radiopharmaceuticals, survival

## Abstract

**Simple Summary:**

This retrospective study explored the progression-free survival (PFS) of patients from Appalachian Kentucky with metastatic, persistent, or recurrent uterine cervix cancer, focusing on outcomes with various lines of chemotherapy. This study analyzed data from 127 patients treated between 2002 and 2023, examining first-, second-, and third-line treatments. After the first-line chemotherapy, the median PFS was 5.5 months. For the second- and third-line treatments, the median PFS was 5.3 and 3.0 months, respectively. Patients receiving cisplatin-containing regimens had a slightly better first-line PFS of 6.5 months. This study suggests that a five-month PFS may serve as a benchmark for future trials evaluating the efficacy of radiopharmaceuticals in this patient population. This study emphasizes the need for new treatment options, as survival outcomes tend to decrease with subsequent lines of therapy.

**Abstract:**

**Background:** Prior preclinical studies showed promising antitumor activity and an acceptable safety profile associated with radiopharmaceuticals for patients with metastatic, persistent, or recurrent uterine cervix cancers. Whether the addition of a radiopharmaceutical to chemotherapy would significantly increase progression-free survival in such patients is untested. Our retrospective study sought to associate the line of treatment and progression-free survival as benchmarks for next-generation radiopharmaceutical development. **Methods:** We grouped metastatic, persistent, or recurrent uterine cervix cancer patients not amenable to curable surgery or radiotherapy between 2002 and 2023 by the line of doublet, triplet, and quadruplet chemotherapy or another intervention. After the first-line treatment, patients were monitored for radiographic progression every three months for up to three years. The primary endpoints were the first and any second or third progression-free survival intervals. **Results:** A total of 127 patients contributed demographic, tumor, line of treatment, and outcome data with a median follow-up of 18 months (25–75% interquartile range: 9 to 37 months). After the first-line treatment, 113 patients had local or distant progression or died from any cause, most often death from the disease (67%). Median progression-free survivals were 5.5 months (95% confidence interval: 4.8–6.0 months), 5.3 months (95% confidence interval: 4.5–6.3 months), and 3.0 months (95% confidence interval: 2.1–3.7 months) for the first-, second-, and third-line treatments, respectively. For a first-line cisplatin-containing regimen, the median progression-free survival was 6.5 months (95% confidence interval: 5.5–7.7 months). **Conclusions:** This study highlights the limited efficacy of current treatments for metastatic, persistent, or recurrent uterine cancer patients. A five-month progression-free survival might serve as a benchmark for the development of novel therapies in clinical efficacy trials, such as radiopharmaceuticals.

## 1. Introduction

Uterine cervix cancer remains a serious health threat, with an estimated 604,000 new cases diagnosed worldwide in 2020, which makes it the fourth most common malignancy among women [1]. Even in the United States, the estimated incidence and mortality were 13,960 and 4310, respectively, in 2023 [2]. In their earliest stages, advanced uterine cervix cancers are preventable with screening or vaccination, or, in general, are curable by surgery. In their advanced stages, pelvic radiotherapy and concurrent cisplatin chemotherapy followed by brachytherapy provide durable responses and many cures [3,4,5]. Metastatic, persistent, or recurrent disease not amenable to radical surgery or to regional radiotherapy is managed by cytotoxic or biologic (immuno)chemotherapy. There are now eight landmark phase II or III trials in the metastatic, persistent, or recurrent disease clinical setting, with two multi-agent cisplatin-containing combinations being superior to single-agent cisplatin given at 50 mg m^−2^ every three weeks [6,7,8,9,10,11,12,13]. When added to cisplatin, paclitaxel at 175 mg m^−2^ and bevacizumab at 15 mg kg^−1^ on a 21-day cycle extended median progression-free survival to eight months [11]. Adding pembrolizumab 200 mg every three weeks to the three-drug regimen further prolonged median progression-free survival to 10 months [12]. Although both regimens were associated with adverse events, neither led to a decrement in patient quality of life [14,15].

In the metastatic, persistent, or recurrent uterine cervix cancer clinical setting, it may be unreasonable to recycle cisplatin-containing regimens for patients currently progressing (or previously failing to respond) on a standard agent. Therefore, the development of radiopharmaceuticals, alone or in an anticancer drug combination, and searching for new radiosensitizers make sense [16,17,18]. This retrospective study began as an effort to provide progression-free survival data on cisplatin-containing doublet, triplet, or quadruplet cytotoxic and biologic (immune)chemotherapy. It was intended to provide survival benchmarks for a population living in an urban manufacturing and rural agricultural region that would serve as subjects in early-stage safety and efficacy studies of radiopharmaceuticals [16,17,18,19].

## 2. Materials and Methods

### 2.1. Study Population

**Figure 1:**
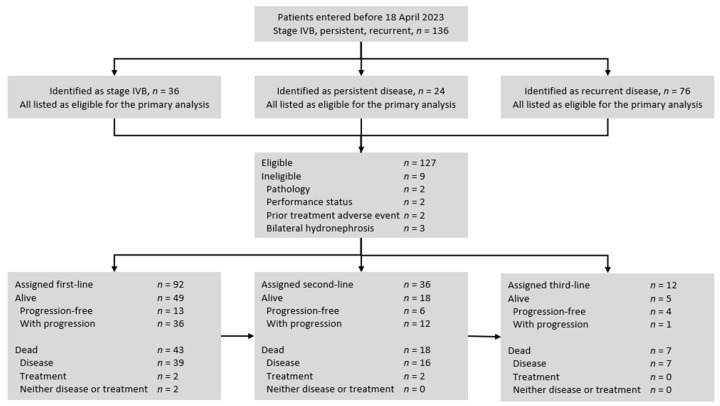
STROBE flow diagram for uterine cervix cancer patients included in this retrospective cohort analysis. Initially, 136 women with metastatic stage IVB, persistent, or recurrent uterine cervix cancer were identified as having received treatment between January 2001 and April 2023. After exclusions, 127 women were grouped by line of treatment and regimen and studied for progression-free survival.

The University of Kentucky Institutional Review Board (Lexington, Kentucky, protocol #84471) approved this retrospective study. Eligible study patients were women with metastatic stage IVB, persistent, or recurrent uterine cervix cancer (Figure 1, Table 1). Histological subtypes included squamous, adenosquamous, and adenocarcinoma cancers. While documentation of the primary uterine cervix cancer was required, a biopsy confirmation of metastatic disease was not required for lesions identified using computed tomography, positron emission tomography, or magnetic resonance imaging if the lesion was more than two centimeters in diameter. In patients with small-volume metastatic disease (<2 cm), biopsy of at least one lesion was required for inclusion in this retrospective study. Eligible patients had an Eastern Cooperative Oncology Group (ECOG) performance status of 0, 1, or 2; had recovered from the effects of any primary cisplatin-based radiochemotherapy (>4 weeks); and had to be free of infection prior to first-line therapy for metastatic, persistent, or recurrent disease. Ineligible patients for this study included those who had a concurrent or past malignancy other than cervical cancer, had a central nervous system metastasis, or had a bilateral hydronephrosis that could not be alleviated by ureteral stents or percutaneous nephrostomy prior to first-line therapy for metastatic, persistent, or recurrent disease. The study population lived in central and eastern Kentucky [19].

### 2.2. Treatments

Cisplatin-based regimens were given by vein and repeated every three weeks: paclitaxel 135 mg m^−2^ plus cisplatin 50 mg m^−2^ or carboplatin area under the receiver operator curve 5; or paclitaxel 175 mg m^−2^, cisplatin 50 mg m^−2^ plus bevacizumab 15 mg kg^−1^ with or without pembrolizumab 200 mg. First-, second-, and third-line treatments are listed in Table 2.

Treatment of primary uterine cervix cancer disease might have included external beam radiotherapy (45 Gy in 25 fractions) followed by intracavitary or interstitial brachytherapy involving low-dose-rate (40 Gy in 1 or 2 fractions) or high-dose-rate (27.5–30 Gy in 5 fractions) prescriptions. The radiotherapy treatment intended a duration not to exceed 56 ± 3 days. Concurrent weekly intravenous cisplatin (40 mg m^−2^, not to exceed 70 mg total per week) was encouraged for a maximum of six cycles [19]. Cisplatin given during definitive radiotherapy was not considered a line of systemic chemotherapy for this analysis.

### 2.3. Assessments

This study was developed as a retrospective analysis of progression-free survival by an investigator who was unaware of the treatment assignments and clinical assessments of disease status when radiographic progression or clinical deterioration prompted a change in treatment. Patients were followed, in general, quarterly for three years, semiannually for the next two years, and then annually until death.

### 2.4. Endpoints

Progression-free survival was defined as the time from (immuno)chemotherapy intervention to disease progression, clinical and radiographic, that resulted in a change in therapy. Local or distant recurrence or death from any cause was determined by an investigator who was unaware of the treatment assignment. For this analysis, salvage surgery or regional radiotherapy did not contribute to the progression-free survival calculations. Progression-free survival intervals were calculated for up to three lines of treatment. After patients completed a line of therapy, the reason for a change in or discontinuation of treatment as determined by local treating oncologists was documented and expressed as a proportion of total events observed for that line of therapy.

### 2.5. Statistical Analyses

Progression-free survival was assessed in an intent-to-treat population, which included all patients receiving (immuno)chemotherapy. Patients for whom there were no results with respect to disease status or missing data were considered not to have had a disease progression event. The Kaplan–Meier method was used to estimate progression-free survival. Treatment differences between lines of therapy were not assessed by the log-rank test due to having too few treated patients. Data were compiled using Microsoft Excel (version 16.67). For the ease of comparison, null values (i.e., historical progression-free survival) for cisplatin doublet, triplet, or quadruplet (immuno)chemotherapy in the metastatic, persistent, or recurrent uterine cervix cancer treatment setting are listed in Table 3.

## 3. Results

### 3.1. Patient Characteristics

From November 2002 to April 2023, 136 patients were identified with metastatic (untreated stage IVB), persistent, or recurrent uterine cervix cancers (Figure 1). The primary analyses excluded a total of nine patients, which included two patients for an atypical pathology (small cell or neuroendocrine), two patients for a performance status greater than ECOG 2 (from central nervous system metastases), two patients for a prior treatment adverse event precluding the first-line treatment, and three patients for a bilateral hydronephrosis that could not be alleviated by ureteral stents or percutaneous nephrostomy. No patients were found to be ineligible for having a concurrent or past malignancy. After the exclusions, 127 (93%) patients were evaluable for progression-free survival interval(s). Of the 127 patients, 120 (94%) had completed prior cisplatin and radiotherapy for the primary disease; 22 (18%) had persistent primary uterine cervix cancer identified in a previously irradiated field. Patient, tumor, and treatment characteristics are summarized in Table 1. Treatments administered during the study period are listed in Table 2. Median follow-up was 18 months (25–75% interquartile range: 9 to 37 months).

### 3.2. Progression-Free Survival

With 113 events occurring after the first-line treatment, Kaplan–Meier estimates of the median progression-free survival for patients who received (immuno)chemotherapy were 5.5 months (95% confidence interval, 4.8 to 6.0 months). The most common event was disease progression associated with death (67%). First-line cisplatin-containing treatment involved either a cisplatin/paclitaxel/bevacizumab triplet (39%) or another platinum doublet (25%) most often. The median progression-free survival for the first-line cisplatin-containing treatment was 6.5 months (95% confidence interval, 5.5 to 7.7 months).

With an additional 38 events observed after the second-line chemotherapy, Kaplan–Meier estimates of the median progression-free survival for patients were 5.3 months (95% confidence interval, 4.5 to 6.3 months). The most common event was disease progression associated with death (68%). The second-line treatment involved a platinum combination (19%) less often than in the first-line treatment.

After the third-line treatment, there were an additional nine events, six of which (67%) were due to disease progression resulting in death. A Kaplan–Meier estimate for median progression-free survival for the third-line chemotherapy treatment was 3.0 months (95% confidence interval, 2.1 to 3.7 months). The third-line treatment rarely involved a platinum combination (8%).

## 4. Discussion

In this retrospective study involving patients with metastatic, persistent, or recurrent uterine cervix cancer, the first progression-free survival interval was 5.5 months after (immuno)chemotherapy. The benefit of a first-line cisplatin-containing combination was marginal, with an index disease progression associated with death as the most common event. This finding differs from the results of the Gynecologic Oncology Group (GOG) GOG240 trial [11], which showed a sustained survival benefit of bevacizumab added to a cisplatin–paclitaxel chemotherapy doublet (8.2 versus 6.0 months, *p =* 0.0002). So too were our results different from the findings of the KEYNOTE-826 trial [12], where the addition of pembrolizumab to the cisplatin–paclitaxel–bevacizumab regimen returned a median two-month gain in progression-free survival (10.4 versus 8.2 months, *p* < 0.001). Our inconsistent results may be related to the treating physician’s discretion for chemotherapy regimen over the study period, the disease status (preponderance of recurrent rather than metastatic or persistent disease), socioeconomic factors impacting treatment and timely follow-up, or all of these factors [19].

The present results are consistent with findings from previous studies of cisplatin doublets for the treatment of metastatic, persistent, or recurrent uterine cervix cancer (Table 3). In the phase III GOG0169 study of cisplatin plus paclitaxel for metastatic, persistent, or recurrent uterine cervix cancer, the median progression-free survival was 4.8 months [8]. In the phase III GOG0179 study of cisplatin plus topotecan, the median progression-free survival was 4.6 months [9]. A similar benefit was observed in a phase III trial of four cisplatin-containing doublets where the median progression-free survivals were 5.8 months for cisplatin–paclitaxel, 3.9 months for cisplatin–vincristine, 4.7 months for gemcitabine–cisplatin, and 4.6 months for cisplatin–topotecan [7]. Taken together, these results suggest that first-line platinum-containing doublet chemotherapies may provide a progression-free survival benefit of about five months in those who have metastatic, persistent, or recurrent uterine cervix cancer. Our findings suggest that second-line treatments confer a similar five-month median survival interval, but this survival interval diminishes with subsequent lines of therapy. Based on these observations, a five-month benchmark for the progression-free survival interval for trials investigating radiopharmaceuticals for the treatment of metastatic, persistent, or recurrent uterine cervix cancer seems reasonable.

Several potential sources of bias must be addressed in studies like ours, where progression-free survival is the endpoint of interest. Unlike overall survival, which can be objectively determined by the known or recoverable timing of death, disease progression involves subjective assessments that can vary. One type of bias, evaluation-time bias, arises from differences in the timing of disease assessments between treatments. In our study, we minimized this bias by using an independent investigator, uninvolved in patient treatment, to assess progression, thereby avoiding clinical subjectivity related to treatment changes. Another source of bias is attrition bias, which occurs when patient dropout rates differ by treatment line. This bias is particularly challenging in progression-free survival studies because, unlike the time of death, which is usually recoverable, a patient’s disease status becomes difficult to determine once they are lost to follow-up. A third bias stems from the inconsistency and subjectivity of radiographic imaging. Assessments depend on the individual interpreting the image (e.g., subjectivity in determining the progression of small lesions) and the timing of the imaging (e.g., intervals of 8 weeks vs. 6 months). Although we used a blinded investigator to evaluate the radiographic disease status, we could not control for imaging intervals due to the retrospective nature of our study. Furthermore, studies like ours typically include death as part of the progression-free survival analysis. However, this introduces complications when patients are lost to follow-up for radiologic assessments, and the time of death is recorded significantly after the last imaging exam [20]. In such cases, the interval between the last radiographic evaluation and death may not accurately reflect disease progression, as progression could have occurred much earlier. To reduce this bias, clinical trials could specify a maximum allowable time interval between the last scan and death, ensuring that death is still considered a valid event in the progression-free survival analysis [20].

Due to the limited efficacy of current regimens, newer agents are needed to improve outcomes for these patients, and investigations are underway. The EMPOWER CERVICAL-1 study was a phase III randomized trial that investigated the addition of the monoclonal antibody Cemiplimab after first-line platinum-based chemotherapy for patients with relapsed or metastatic cervical cancer; the median OS was longer with Cemiplimab compared to the investigator’s choice of chemotherapy (12.0 months vs. 8.5 months) [21,22]. Another second-line therapy includes tisotumab vedotin-tftv, a tissue-factor-directed antibody and microtubule inhibitor drug that showed clinically meaningful and durable antitumor activity [23]. Additionally, the use of the human epidermal growth factor 2 (HER2)-directed antibody–drug conjugate Trastuzumab deruxtecan has shown efficacy in pre-treated patients with HER-2 expressing tumors, including cervical cancer [24]. Given that radiation in combination with chemotherapy is efficacious against locally advanced cervical cancer and radiation is the standard of care for FIGO State IB3 -IVA [5,25,26,27], well-targeted radiopharmaceutical therapy may provide the therapeutic advantage needed to improve outcomes for this patient population.

In summary, platinum-containing chemotherapy regimens resulted in a median five-month progression-free survival among patients with metastatic, persistent, or recurrent uterine cervix cancer. The benefit we observed with respect to first- or second-line treatments was consistent across patients. A five-month PFS may serve as a benchmark for future trials evaluating the efficacy of novel therapies, such as radiopharmaceuticals, in this patient population. Analyses of molecular biomarkers that might predict a clinical response to treatment are ongoing in our phase 0 uterine cervix cancer trial (NCT05462951).

## 5. Conclusions

This retrospective study provides valuable insights into the PFS of patients with metastatic, persistent, or recurrent uterine cervix cancer treated with various chemotherapy regimens. The results align with previous studies, suggesting that a median PFS of five months serves as a reasonable benchmark for first-line chemotherapy in these patients.

Importantly, this study highlights the limited efficacy of current treatment options in the later lines of therapy, which underlines the urgent need for novel therapeutic approaches. Given the variability in patient treatment regimens, disease progression patterns, and socioeconomic factors impacting care, further prospective studies are needed to improve survival for this patient population.

A five-month PFS benchmark may serve as a useful metric for evaluating the efficacy of novel therapies, such as radiopharmaceuticals, in clinical trials.

## Figures and Tables

**Table 1:** Demographic, tumor, and treatment characteristics of patient population (*n* = 127).

Characteristic	Number	Mean ± SD	%
Age, years	127	53 ± 13	100
Residence in Appalachian Kentucky	77		61
Race			
White	119		94
Black or African American	7		5
Asian/Pacific	1		1
Ethnicity			
Hispanic	5		4
Non-Hispanic	122		96
Performance status			
0	77		61
1	32		25
2	18		14
Cell Type			
Squamous	91		72
Adenosquamous	10		8
Adenocarcinoma	26		20
Tumor Grade			
1	2		2
2	32		25
3	93		73
Primary tumor size, centimeters		6.0 ± 1.4	
<6 cm	54		43
≥6 cm	73		57
Pelvic nodal metastasis at diagnosis	84		66
Para-aortic nodal metastasis at diagnosis	50		39
Prior primary cisplatin and radiation	120		94
Duration of primary radiochemotherapy, days		56 ± 19	
<60	65		54
≥60	55		46
Uncorrected creatinine pretherapy, mg dL^−1^			
≥2.0	11		9
1.5–2.0	6		4
<1.5	110		87

Abbreviations: SD = standard deviation of the mean.

**Table 2:** Treatment regimens administered in the first-, second-, or third-line.

	Treatment Line, *n* (%) *		
Regimen	First (*n* = 92)	Second (*n* = 36)	Third (*n* = 12)
Cis + Pac + Bev + Pem	4 (4)	0 (0)	0 (0)
Cis + Pac + Bev	36 (39)	4 (11)	1 (8)
Cis + Pac	13 (14)	0 (0)	0 (0)
Carbo + Pac	10 (11)	3 (8)	0 (0)
Another doublet	3 (3)	2 (6)	2 (16)
Pem	3 (3)	7 (19)	3 (25)
Bev	0 (0)	2 (6)	0 (0)
Other singlet	0 (0)	4 (11)	1 (8)
Radiotherapy	9 (10)	6 (17)	5 (42)
Surgery	14 (15)	8 (22)	0 (0)

Abbreviations: Bev = bevacizumab, Carbo = carboplatin, Cis = cisplatin, Pac = paclitaxel, and Pem = pembrolizumab. * may not total 100% due to rounding.

**Table 3:** Treatment regimen, historical overall response rate, and progression-free survival.

Trial (Reference)	Year Started	Stage	Histology	Number	Treatment	Response Rate (%)	Progression-Free Survival (Months)
KEYNOTE-826 [13]	2018	IVB, R, P	SQ, AS, A	308	Cis + Pac + Bev + Pem	68	10.4 (95% CI: 9.7–12.3)
KEYNOTE-158 [14]	2016	IVB, R, P	SQ, AS, A	98	Pem	12	2.1 (95% CI: 2.0–2.2)
GOG-0240 [12]	2009	IVB, R, P	SQ, AS, A, O	115	Cis + Pac + Bev	50	8.2 (95% CI: NR)
GOG-227C [11]	2002	R, P	SQ, AS	46	BEV	11	3.4 (95% CI: 2.5–4.5)
GOG-0169 [9]	1997	IVB, R, P	SQ	129	Cis + Pac	36	4.8 (95% CI: NR)
GOG-0204 [8]	2003	IVB, R, P	SQ, AS, A	103	Cis + Pac	29	5.8 (95% CI: 4.5–7.6)
				108	Cis + Vin	26	3.9 (95% CI: 3.2–5.2)
				112	Cis + Gem	22	4.7 (95% CI: 3.6–5.6)
				111	Cis + Topo	23	4.6 (95% CI: 3.7–5.8)
GOG-0179 [10]	1999	IVB, R, P	SQ, AS, A, O	147	Cis + Topo	27	4.6 (95% CI: NR)
GOG-0049 [7]	1978	IVB, R, P	SQ	150	Cis 50	21	3.7 (95% CI: NR)
				166	Cis 100	31	4.6 (95% CI: NR)
				128	Cis 20 × 5	25	3.9 (95% CI: NR)

Abbreviations: A = adenocarcinoma, AS = adenosquamous, BEV = bevacizumab, CI = confidence interval, Cis = cisplatin, Gem = gemcitabine, GOG = Gynecologic Oncology Group, IVB = metastatic, NR = not reported, O = other, P = persistent, Pac = paclitaxel, Pem = pembrolizumab, R = recurrent, SQ = squamous cell, Topo = topotecan, and Vin = vinorelbine.

## Data Availability

The raw data supporting the conclusions of this article will be made available by the authors without undue reservation.
